# Supplementary material for: CXCR6+ CD127– Tr1 cells balance immunity and persistence in Plasmodium falciparum infection
Source: J Clin Invest. 2026 May 5;136(14):e200628. doi: 10.1172/JCI200628 (PMC13367961; doi:10.1172/JCI200628)
Supplement: Supplemental data [file jci-136-200628-s168.pdf]

Supplementary Materials for: CXCR6^{+} CD127^{-} Tr1 Cells Balance Immunity and Persistence in Plasmodium falciparum Infection

- Supplemental Figures
  - Supplemental Figures 1 – 21
- Supplemental Tables
  - Supplemental Tables 1 – 2

Supplemental Figures

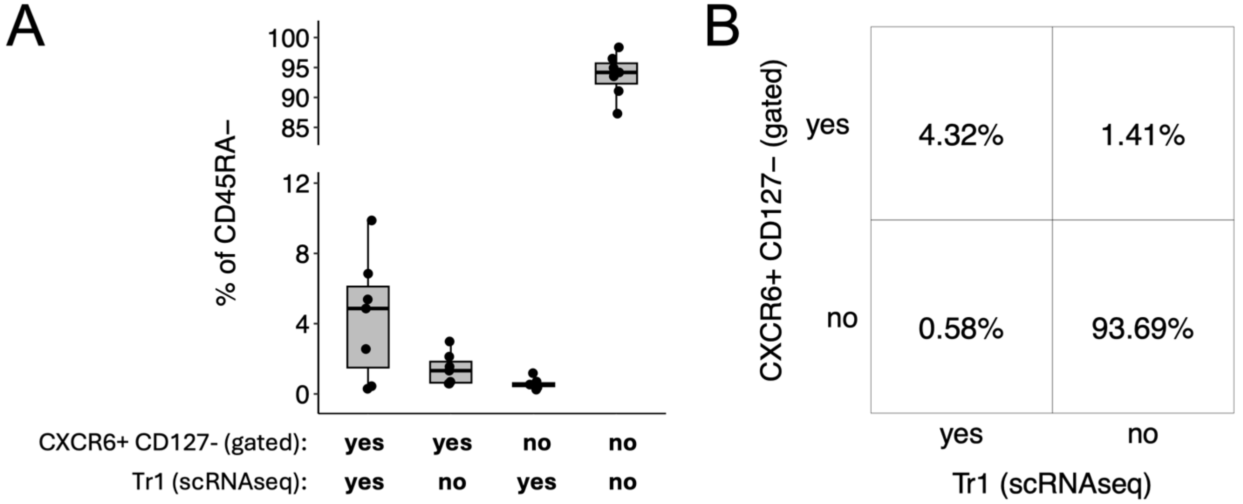

Supplemental Figure 1. Confusion matrix (and underlying data) describing the effectiveness of CXCR6+ CD127- as a gating strategy for identifying Tr1 cells. (**A**) Box plots displaying the percent of CD45RA- (memory) CD4+ T cells that are Tr1 or non-Tr1 and that fall inside or outside of the CXCR6+ CD127- gate. A break in the y-axis is used to improve data visualization. (**B**) Confusion matrix reporting averages of the percentages in 'A'. Sensitivity of the gating strategy can be calculated by dividing true positives (upper-left quadrant) by all positives and multiplying by 100%. Specificity can be calculated by dividing true negatives (bottom-right quadrant) by all negatives and multiplying by 100%.

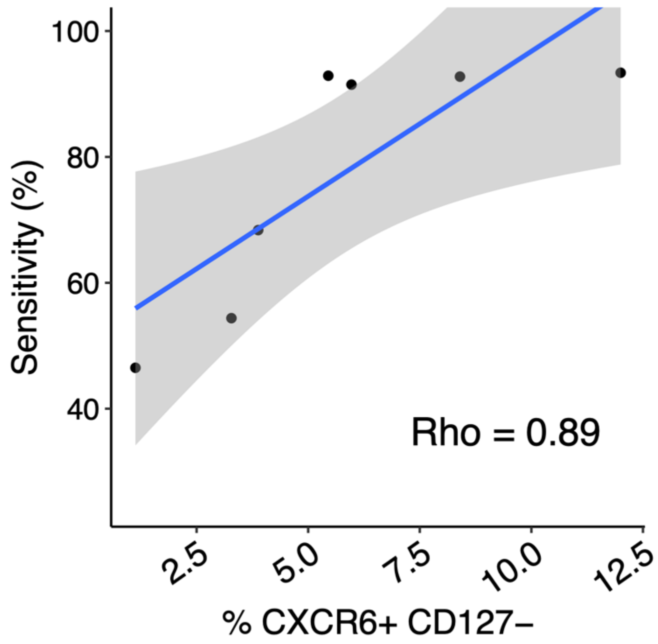

Supplemental Figure 2. Spearman correlation between CXCR6+ CD127- cell abundance and gating strategy sensitivity. Spearman's rho is displayed, and the shaded region represent the 95% confidence range.

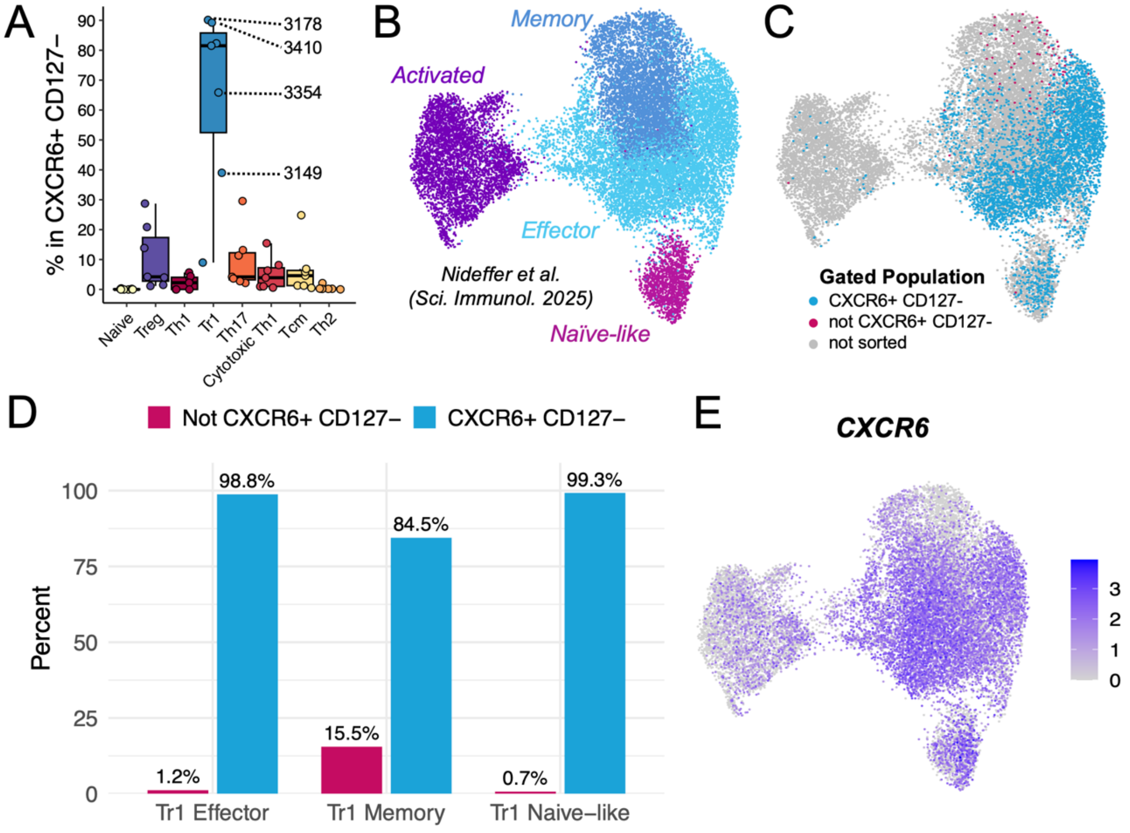

Supplemental Figure 3. Quantification of the heterogeneity amongst CXCR6+ CD127- memory CD4+ T cells. (**A**) The percentage of CXCR6+ CD127- memory CD4+ T cells that belong to different subsets (defined by scRNAseq). Numbers indicate which data points correspond to each of the four donors. (**B**) UMAP from our prior study depicting the heterogeneity amongst Tr1 cells. (**C**) The same UMAP as in 'B' colored according to whether or not cells were sorted as CXCR6+ CD127-. (**D**) Bar graph quantifying the percentage of a given Tr1 cell subset that was captured (or not captured) by the CXCR6+ CD127- gate. (**E**) The same UMAP as in 'B' colored according to expression of CXCR6.

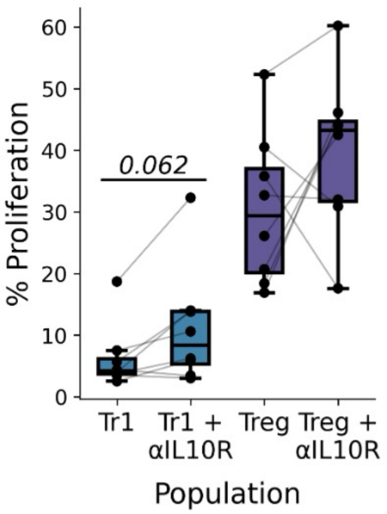

Supplemental Figure 4. Tr1 and conventional Treg proliferation in the presence of IL-10 receptor blockade. Cells were incubated for 4 days in the presence of aCD3 and aCD28 as part of a suppression assay. Lines connect different conditions using cells that that were derived from the same donor. Paired T tests were performed comparing conditions with and without IL-10 receptor blockade.

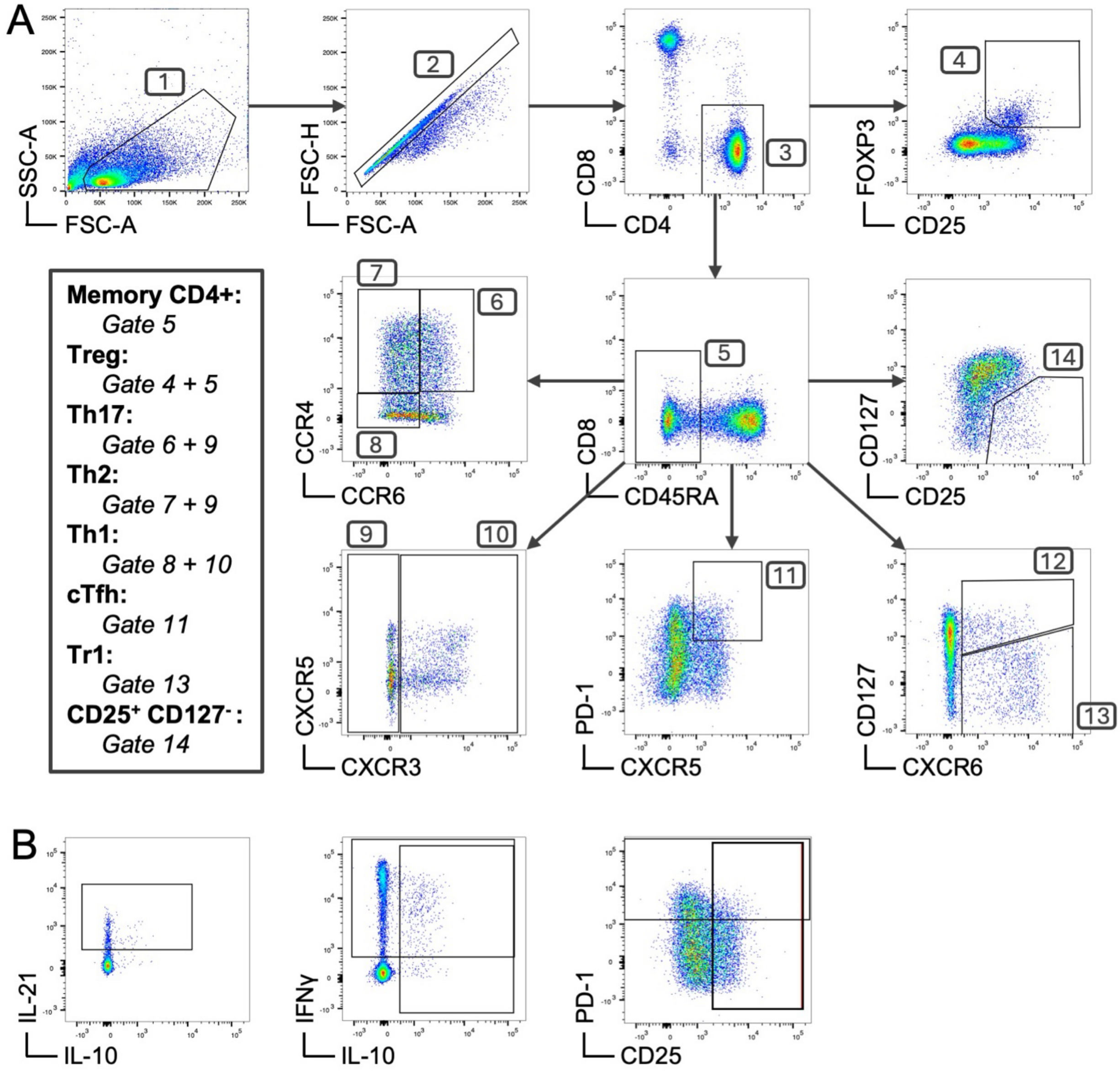

Supplemental Figure 5. Gating of CD4+ T cell subsets and effector molecules. (**A**) Flow cytometry plots depicting the gating strategy for identifying different cellular populations. Population and gate associations are listed in the legend on the left. Gate 12 represents a population of CXCR6+ CD127+ memory CD4+ T cells that were not analyzed in the present study. (**B**) Flow cytometry plots showing staining of key cytokines and surface-expressed effector molecules.

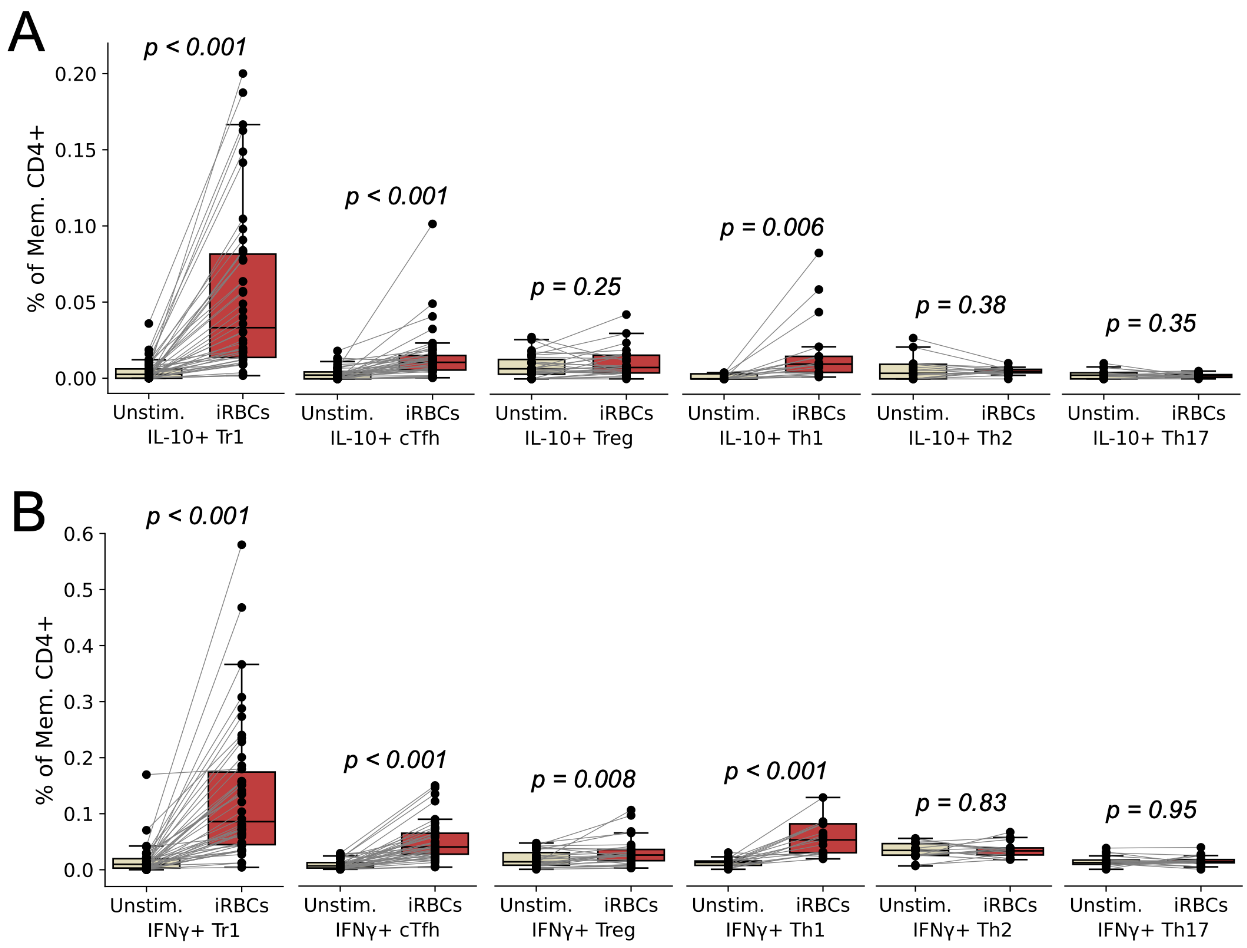

Supplemental Figure 6. Cytokine upregulation by surface-marker defined CD4+ T cell subsets in response to *Plasmodium* antigen stimulation. (**A,B**) The percentage of ('A') IL-10-expressing and ('B') IFNγ-expressing populations in unstimulated versus iRBC-stimulated conditions. (**C,D**) The percentage of ('C') IL-10-expressing and ('D') IFNγ-expressing populations of CD25+ CD127- "Tregs" in unstimulated versus iRBC-stimulated conditions. (**E**) Expression of IL-21 by surface marker-defined Tr1 and cTfh populations in unstimulated versus iRBC-stimulated conditions.

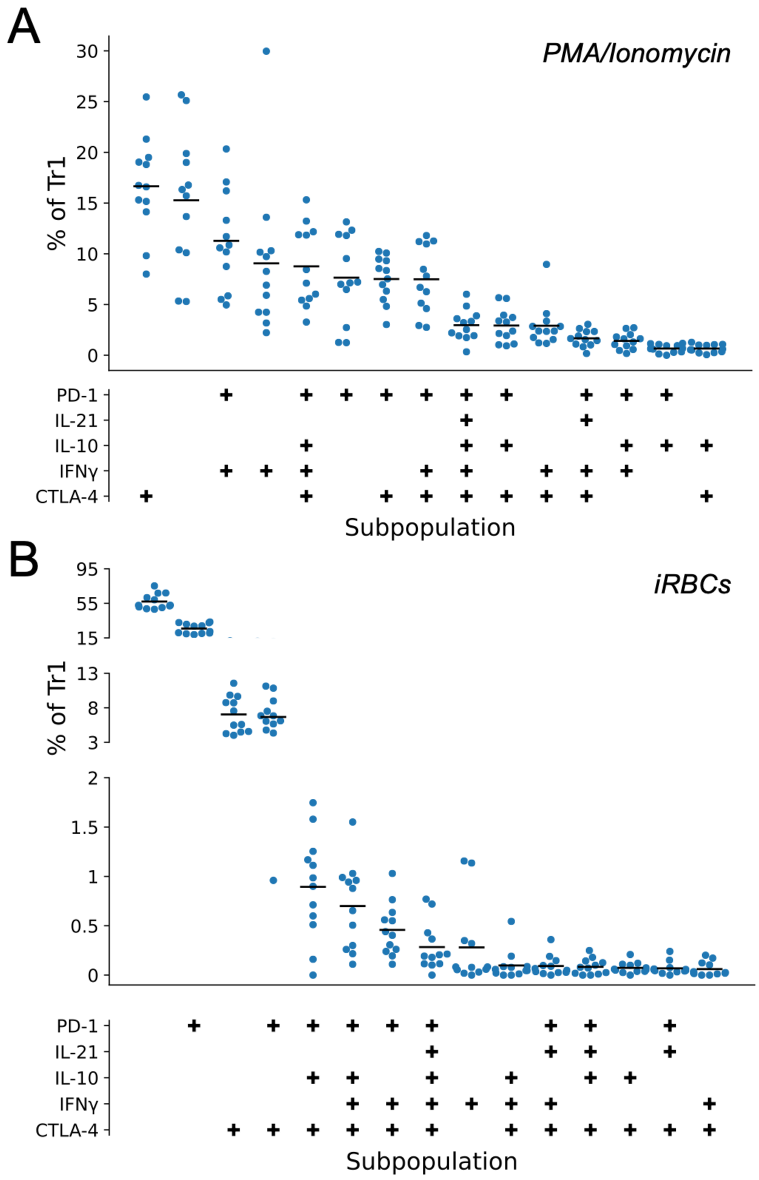

Supplemental Figure 7. Protein expression by CXCR6+ CD127- Tr1 cells following stimulation. (**A-B**) PBMCs from malaria-exposed Ugandans were stimulated with either PMA and Ionomycin (‘A’) or *Plasmodium falciparum*-infected red blood cells (iRBCs) (‘B’), and then, the expression of various proteins was quantified among CXCR6+ CD127- Tr1 cells. Only the 15 most prevalent subpopulations are shown for each stimulation condition. Each data point represents the aggregate of repeated measurements across timepoints for a given study participant.

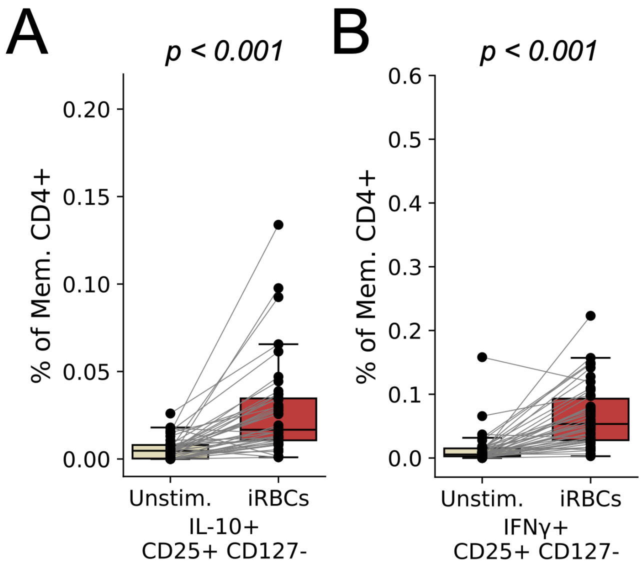

Supplemental Figure 8. Cytokine upregulation by CD25+ CD127- "Tregs" in response to *Plasmodium* antigen stimulation. (**A,B**) The percentage of ('A') IL-10-expressing and ('B') IFNγ-expressing populations of CD25+ CD127- "Tregs" in unstimulated versus iRBC-stimulated conditions.

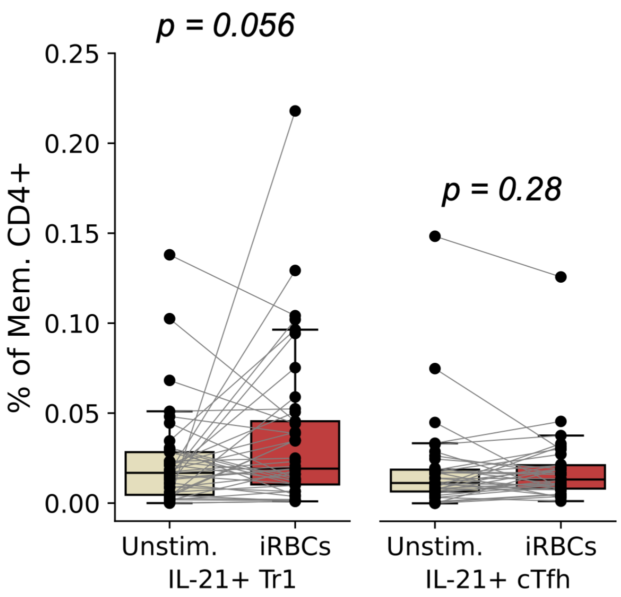

Supplemental Figure 9. Expression of IL-21 by surface marker-defined Tr1 and cTfh populations. Percentage of memory CD4+ T cells gated as IL-21-expressing Tr1 (left) or cTfh (right) in unstimulated versus iRBC-stimulated conditions.

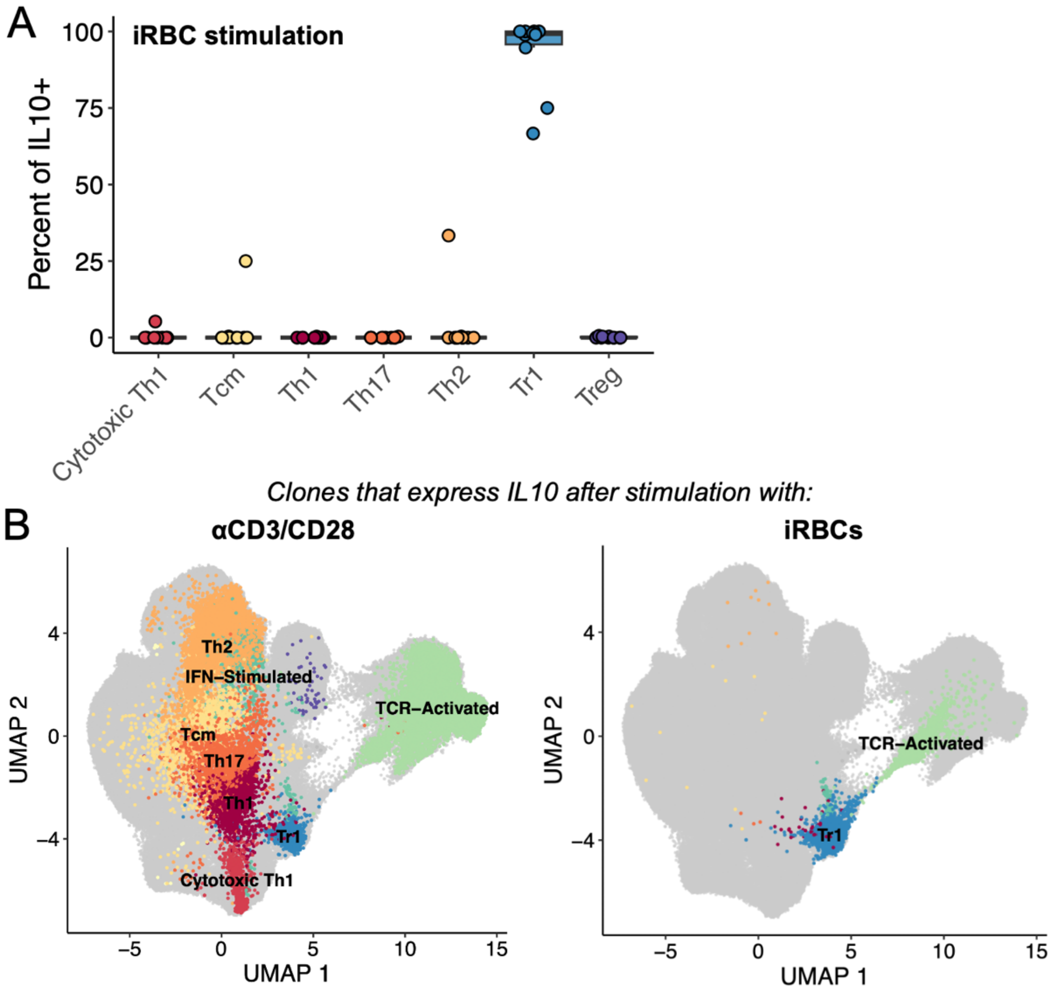

Supplemental Figure 10. Identity of CD4+ T cells that express *IL10* transcripts in response to *Pf* antigen stimulation. (**A**) The percentage of *IL10*-expressing CD4+ T cells (after stimulation with iRBCs) that belong to each of seven memory and effector subsets. (**B**) Cells belonging to a clonotype that was observed to express *IL10* in response to stimulation with αCD3/CD28 beads (left) or iRBCs (right). Cells are colored according to subset identity if *IL10* was detected; light gray cells belonged to a clonotype that was not observed to express *IL10* in response to the stated stimulation conditions.

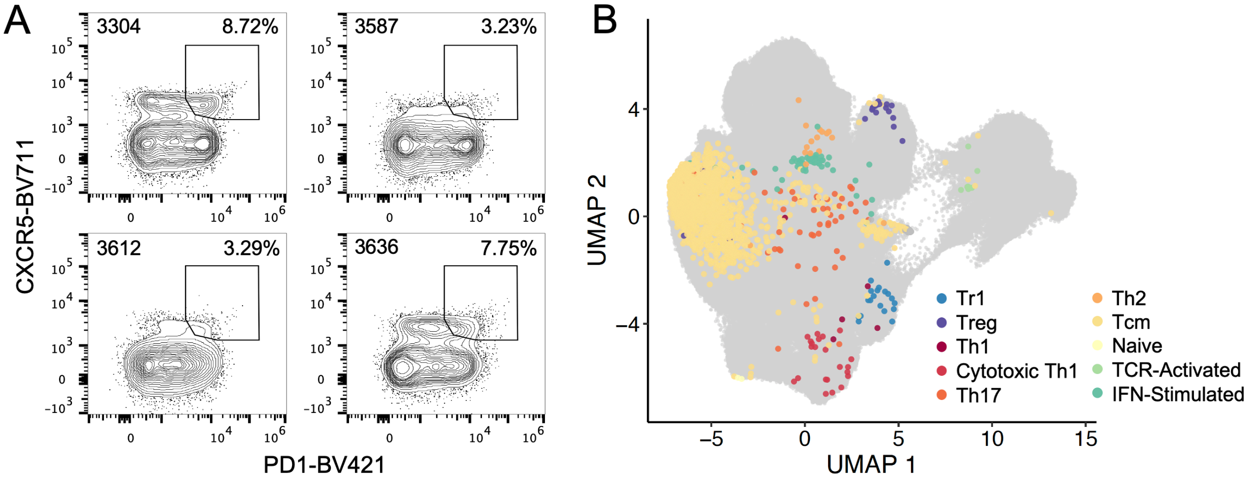

Supplemental Figure 11. Sorting and sequencing of CD4_ T cells co-expressing CXCR5 and PD-1. (**A**) Flow cytometry plots depicting populations from four different Ugandan children that were sorted and then analyzed by scRNAseq. (**B**) Reference mapping of the populations sorted in 'A' to assign annotations and determine the identity of CXCR5+ CD127- cells. Sorted cells are colored according to their annotated identity; cells colored in gray were not sorted and serve only to convey the UMAP architecture.

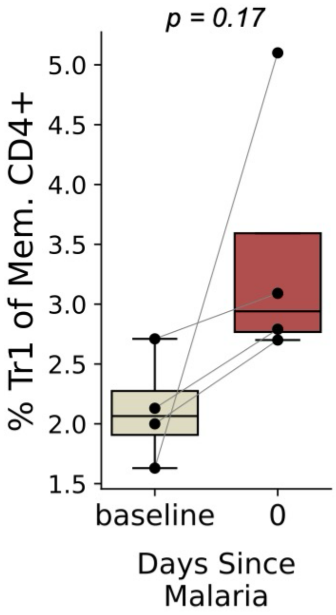

Supplemental Figure 12. Expansion of Tr1 cell frequencies in Ugandan adults diagnosed with malaria. Tr1 frequencies were measured as a proportion of memory CD4+ T cells at a baseline timepoint prior to infection and at the time of diagnosis. Significance was determined via a paired T test.

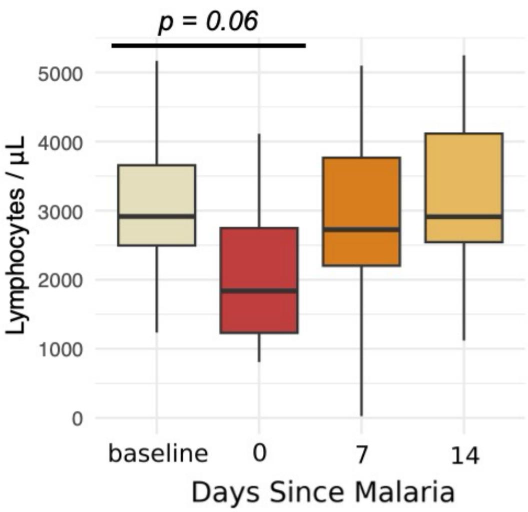

Supplemental Figure 13. Absolute lymphocyte counts in the context of malaria. Lymphocyte counts were measured before, during, and after symptomatic malaria. Significance was determined using a paired T test comparing mean counts at baseline compared to day 0.

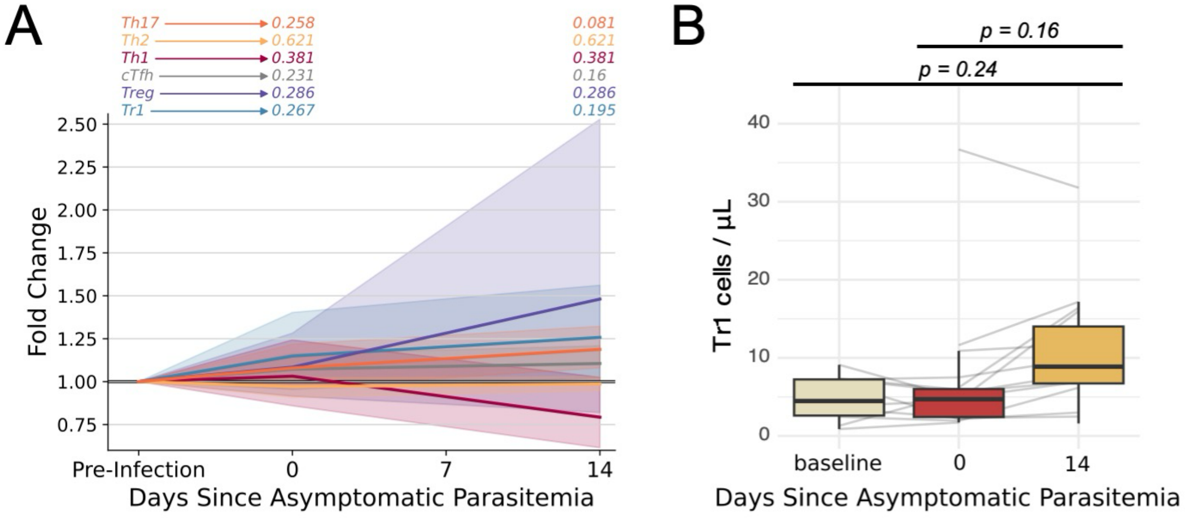

Supplemental Figure 14. Infection dynamics of memory CD4+ T cells populations during asymptomatic parasitemia. (**A**) Fold change (compared to the pre-infection baseline) in cell frequencies following the detection of asymptomatic parasitemia as determined by flow cytometry. P-values are displayed above each sample timepoint for each population and represent pair-wise comparisons to baseline. (**B**) The absolute counts of Tr1 cells in peripheral blood before, during, and after asymptomatic parasitemia.

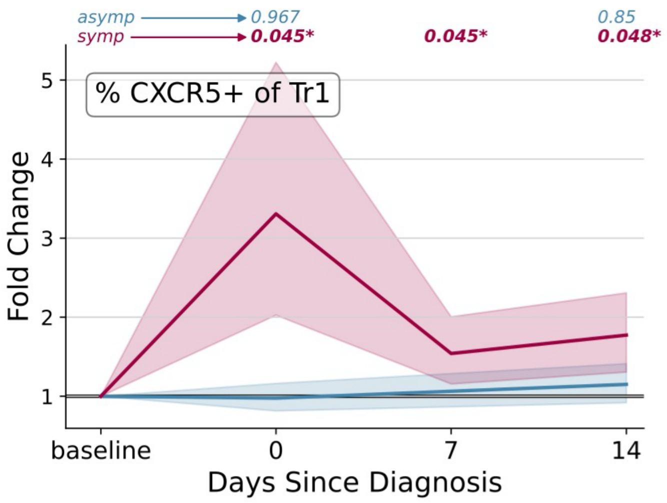

Supplemental Figure 15. Fold change in the percentage of Tr1 cells expressing CXCR5 following symptomatic malaria or asymptomatic parasitemia. p-values are displayed above each sample timepoint for each infection type and represent pair-wise comparisons to baseline.

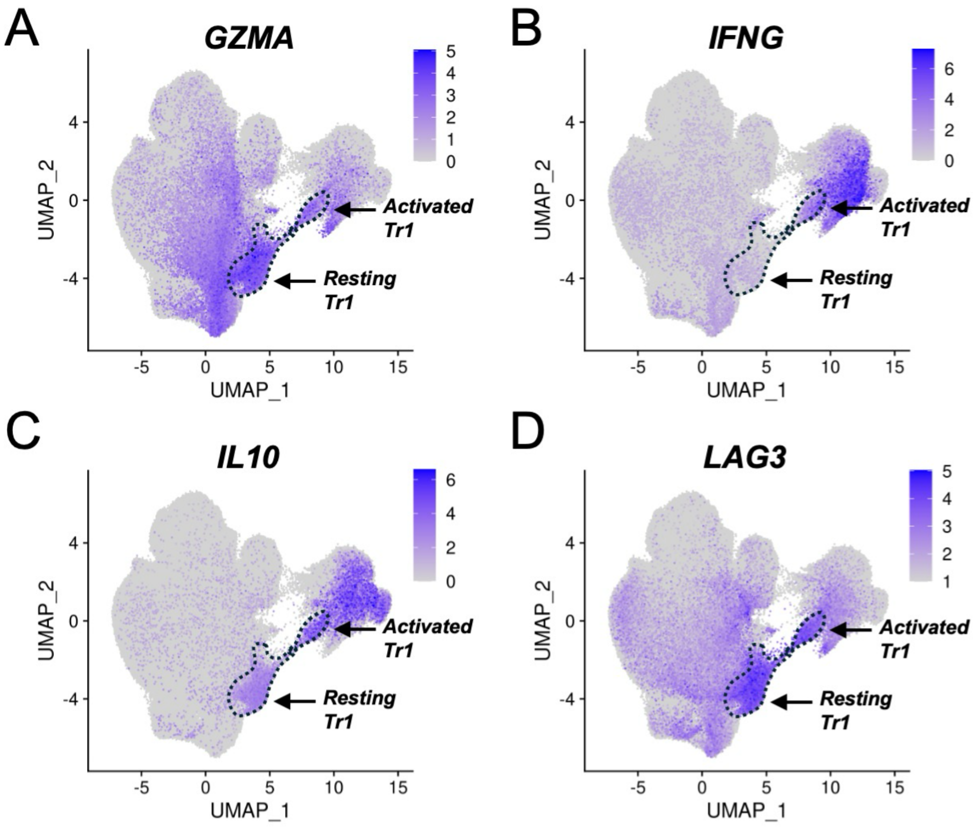

Supplemental Figure 16. Expression of Tr1-associated genes by memory CD4+ T cells. (**A-D**) UMAPs with the same architecture as Fig. 1A colored according to the expression levels of *GZMA* (‘A’), *IFNG* (‘B’), *IL10* (‘C’), and *LAG3* (‘D’). The dashed lines highlight resting and activated Tr1 populations.

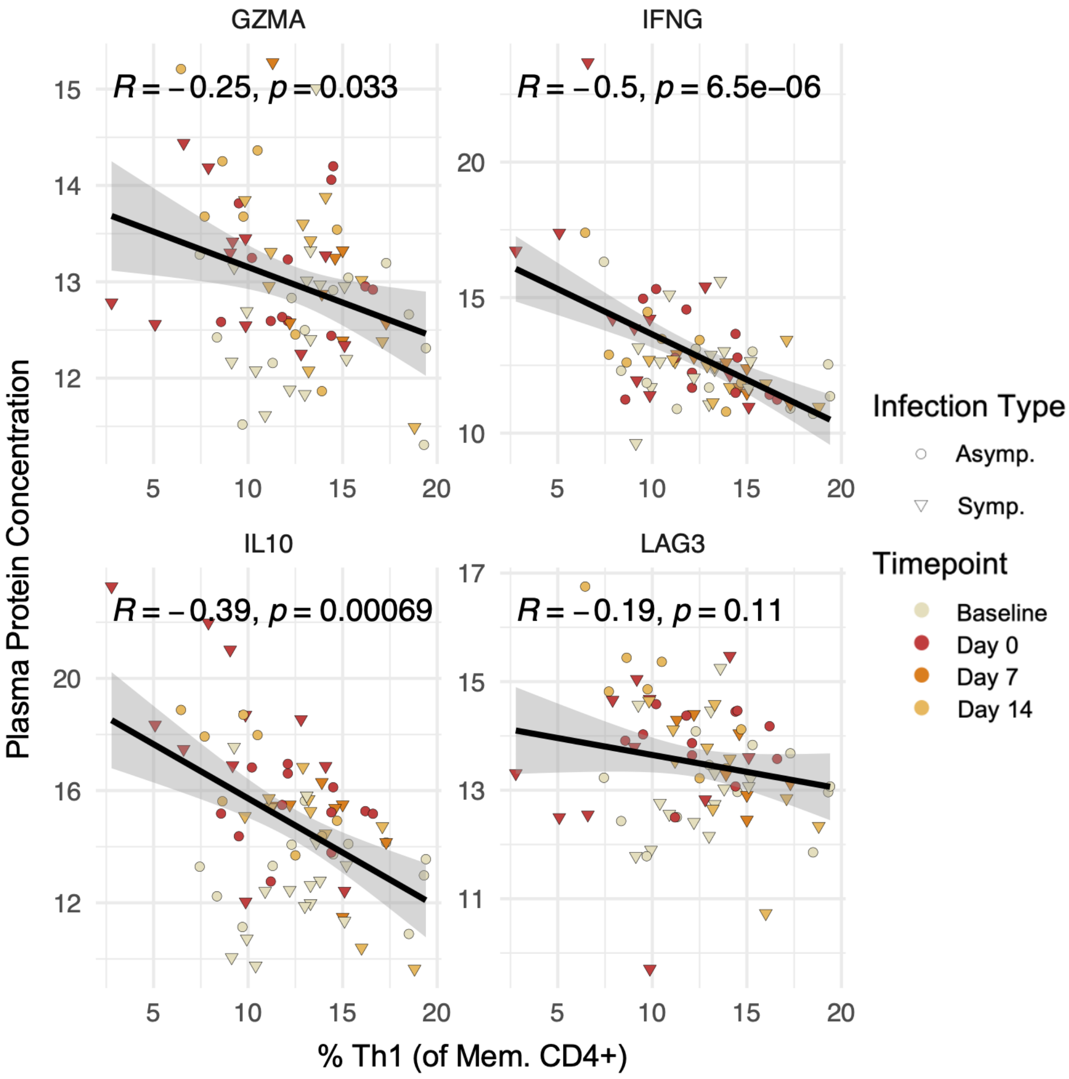

Supplemental Figure 17. Correlations between plasma concentrations of granzyme A, IFNγ, IL-10, and LAG3 (determined by NULISA) and Th1 frequencies (determined by flow cytometry). The color and shape of individual points denote the timepoint of the sample and whether the infection was symptomatic or asymptomatic. Gray area represents 95% confidence range. Pearson's R and associated p-values are displayed above each plot.

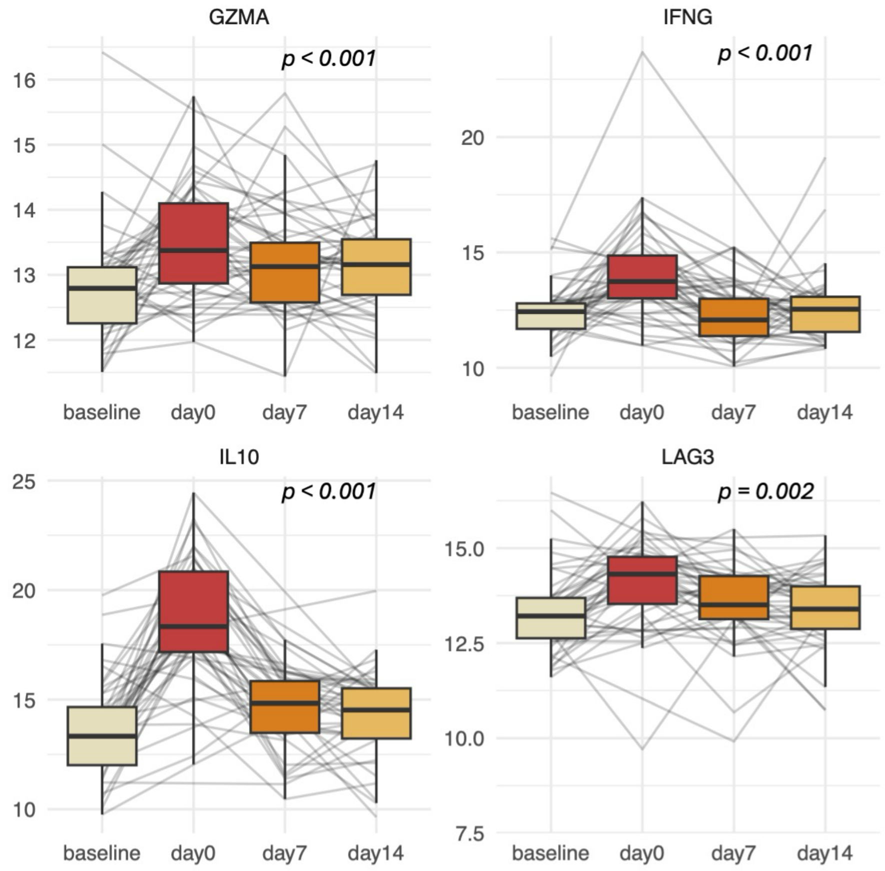

Supplemental Figure 18. Tr1 plasma protein abundance in the context of symptomatic malaria. The abundance of granzyme-A, IFNγ, IL-10, and LAG-3 in plasma samples (determined by NULISA) collected before, during, and after symptomatic malaria or asymptomatic parasitemia. All p-values shown were adjusted to correct for multiple hypothesis testing. Unless the p-value is explicitly reported, p-value < 0.05 = *; p-value < 0.01 = **; p-value < 0.001 = ***.

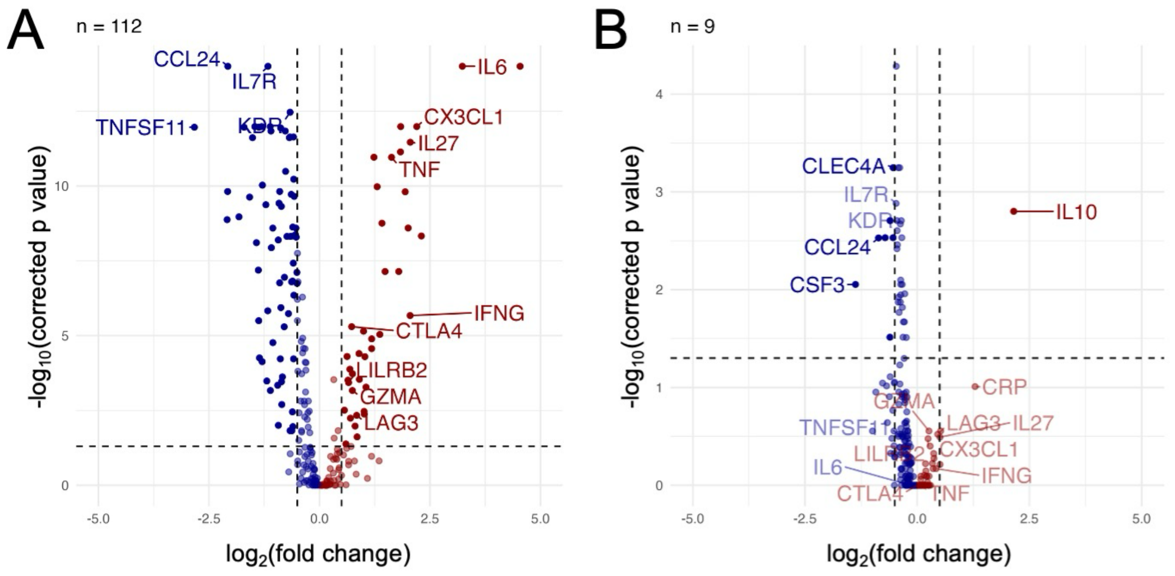

Supplemental Figure 19. Differential abundance of plasma proteins measured by NULISA during symptomatic and asymptomatic infections. (**A-B**) Volcano plots depicting proteins that are differentially abundant at diagnosis with symptomatic malaria ('A') or asymptomatic parasitemia ('B') versus a pre-infection baseline.

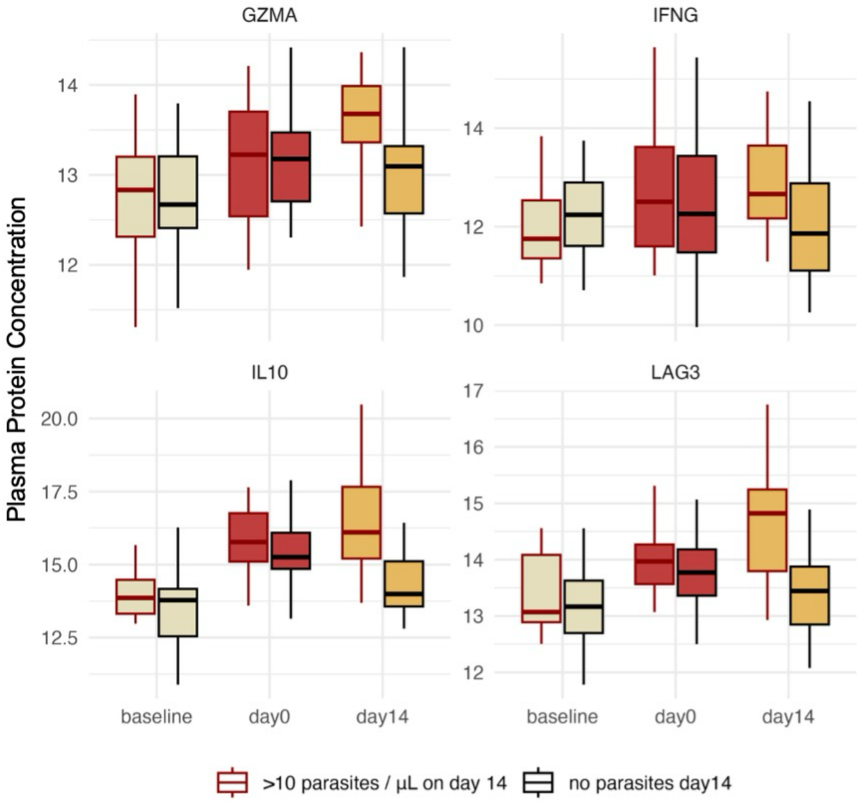

Supplemental Figure 20. The effect of persistent parasitemia on plasma concentrations of Tr1 effector molecules. The abundance of granzyme-A, IFNg, IL-10, and LAG-3 in plasma samples (determined by NULISA) collected before during and after asymptomatic parasitemia, stratified by whether the subject tested parasitemia positive or negative at day 14.

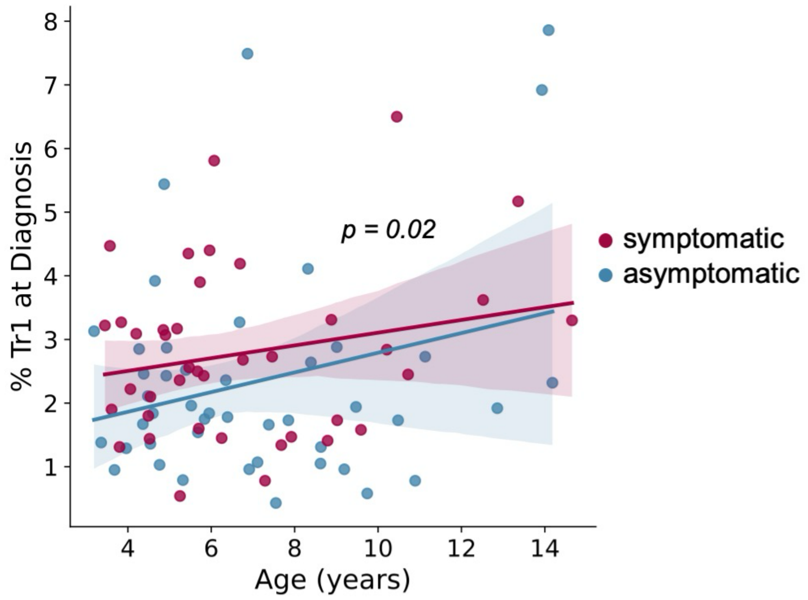

Supplemental Figure 21. Correlation between age and Tr1 frequency at the time of diagnosis. Separate linear regressions are displayed for symptomatic and asymptomatic infections. Shaded regions represent 95% confidence ranges. The displayed p-value represents the significance of an effect of age on Tr1 frequency in a generalized linear model that includes infection type (symptomatic or asymptomatic) as an independent categorical variable.

Supplemental Table 1. Characteristics of MUSICAL study participants.

Characteristic	
**Age in years at enrollment**	4.3 (1.8-13.5)
**Female sex, n (%)**	20 (41.7%)
**Mean parasite density at time of symptomatic infection, SD**	18929.2 (19955.39)
**Mean parasite density at time of asymptomatic infection, SD**	3453.3 (6038.5)
**Sickle cell status***	
**HbAA, n/N(%)**	36/46 (75.0%)
**HbAS, n/N(%)**	10/46 (20.8%)
**Incidence of malaria per person year over period of cohort (2020-2023)**	1.96

*2 individuals with missing Hb genotype data

Supplemental Table 2. Univariate and multivariate analyses of the relationship between Tr1 frequencies and the future incidence of malaria or the duration of incident asymptomatic infections.

	Future incidence of symptomatic malaria over 2 years				Duration of incident asymptomatic infection in days[A]			
	Univariate		Multivariate[B]		Univariate		Multivariate[B]	
	**IRR (95% CI)**	**P**	**IRR (95% CI)**	**P**	**Coef (95% CI)**	**P**	**Coef (95% CI)**	**P**
**Age in years**	0.89 (0.80-0.98)	0.02	0.87 (0.78-0.97)	0.01	0.38 (-3.4-4.2)	0.84	2.2 (-2.5-6.9)	0.36
**Log10 parasite density at diagnosis**	1.06 (0.82-1.37)	0.67	1.02 (0.78-1.32)	0.89	4.15 (-6.9-15.2)	0.46	7.36 (-1.9 – 16.6)	0.12
**%CXCR6+CD127- Tr1 prior to infection**								
**Group 1, n=22 (0.43%-1.5%)**	Ref	Ref	Ref	Ref	Ref	Ref	Ref	Ref
**Group 2, n=40 (1.5%-3.08%)**	1.43 (0.83-2.48)	0.20	1.39 (0.76-2.55)	0.29	23.7 (-3.2-50.6)	0.085	27.3 (-3.5-58.2)	0.08
**Group 3, n=23 (3.1% - 7.86%)**	0.77 (0.37-1.59)	0.48	0.76 (0.37-1.59)	0.47	33.0 (-6.9-3.0)	0.10	42.5 (3.6-81.4)	0.032
**%CXCR6+CD127- Tr1 at time of diagnosis**								
**Group 1, n=22 (0.43%-1.5%)**	Ref	Ref	Ref	Ref	Ref	Ref	Ref	Ref
**Group 2, n=40 (1.5%-3.08%)**	0.74 (0.33-1.62)	0.45	0.74 (0.35-1.57)	0.44	15.5 (-14-45.1)	0.31	11.23 (-21.5-44.0)	0.50
**Group 3, n=23 (3.1% - 7.86%)**	0.38 (0.18-0.80)	0.011	0.38 (0.18-0.80)	0.011	51.8 (17.4-86.3)	0.003	52.67 (18.9-86.5)	0.002

IQR:
[A]Incidence infections defined by AMA1 amplicon sequencing.
[B]Multivariate models adjusting for age in years and log_{10} parasite densities (detected by quantitative PCR)
